# Genomic, Evolutionary, and Pathogenic Characterization of a New *Polerovirus* in Traditional Chinese Medicine *Viola philippica*

**DOI:** 10.3390/v17010114

**Published:** 2025-01-15

**Authors:** Yuanling Chen, Gaoxiang Chen, Jiaping Yu, Yali Zhou, Shifang Fei, Haorong Chen, Jianxiang Wu, Shuai Fu

**Affiliations:** 1 Research Center for Life Sciences Computing, Zhejiang Lab, Hangzhou 311100, China; 2 Hainan Institute, Zhejiang University, Sanya 572025, China; 3 State Key Laboratory of Rice Biology, Key Laboratory of Biology of Crop Pathogens and Insects of Zhejiang Province, Institute of Biotechnology, Zhejiang University, Hangzhou 310058, China

**Keywords:** Violae Herba, *Viola philippica* polerovirus, RNA-seq, novel viruses, recombination, hypersensitive response, pathogenicity determinant

## Abstract

*Viola philippica*, a medicinal herbaceous plant documented in the Chinese Pharmacopoeia, is a promising candidate for research into plant-derived pharmaceuticals. However, the study of newly emerging viruses that threaten the cultivation of *V. philippica* remains limited. In this study, *V. philippica* plants exhibiting symptoms such as leaf yellowing, mottled leaves, and vein chlorosis were collected and subjected to RNA sequencing to identify potential viral pathogens. A novel polerovirus, named Viola Philippica Polerovirus (VPPV), was identified in *V. philippica*. VPPV possesses a linear, positive-sense, single-stranded RNA genome consisting of 5535 nucleotides (nt) and encodes seven highly overlapping open reading frames (ORFs). Two potential recombination events were identified within ORF2, ORF3a, and ORF3, providing insights into the genetic diversity and evolution history of this novel polerovirus. An infectious cDNA clone of VPPV was successfully constructed and shown to infect *Nicotiana benthamiana*. Using a PVX-based heterologous expression system, the VPPV P0 protein was shown to trigger a systemic hypersensitive response (HR)-like reaction in *N. benthamiana*, indicating that P0 functions as the main pathogenicity determinant. These findings contributed to the detection and understanding of pathogenic mechanisms and control strategies for VPPV in *V. philippica*.

## 1. Introduction

*Viola philippica* Cav., an herbaceous perennial plant primarily indigenous to East Asia, has garnered significant attention in botany and pharmacology due to its well-established medicinal properties in traditional Chinese medicine [1,2]. Its efficacy in treating respiratory infections, skin inflammations, and fevers is attributed to its high content of bioactive compounds, such as flavonoids, alkaloids, and saponins, which exhibit notable antimicrobial, anti-inflammatory, and antiviral activities [3,4,5]. These characteristics make *V. philippica* a promising candidate for further research into plant-derived pharmaceuticals. However, while extensive research has focused on the therapeutic potential of *V. philippica*, its role as a host for plant viruses has not been explored.

The identification of novel viruses in plants has been revolutionized by advances in high-throughput sequencing technologies, particularly RNA sequencing (RNA-seq) [6]. As obligate intracellular parasites, viruses depend on host cellular machinery for replication and often exhibit significant genetic variability. This diversity presents major challenges for the detection and identification of previously unknown or highly divergent viral species using traditional methods, such as electron microscopy, serological assays, or polymerase chain reaction (PCR)/reverse transcription-PCR (RT-PCR) [7]. However, high-throughput sequencing has emerged as a powerful tool to overcome these limitations, providing comprehensive insights into plant viromes [8]. In recent years, RNA-seq has played a pivotal role in discovering new viral species in a wide range of plants, including grain crops [9,10,11], medicinal plants [12], and ornamental plants [13]. These discoveries have contributed to a deeper understanding of plant–virus interactions, viral evolution, and the potential threats posed by these novel pathogens to global agriculture.

Poleroviruses belong to the genus *Polerovirus* and have genomes comprising polycistronic, positive-sense, single-stranded RNA, typically ranging from 5.5 to 6.2 kb in size [14]. Their virions are non-enveloped icosahedral particles, 26–34 nm in diameter [14]. In 2021, the *Polerovirus* genus was reclassified from the family *Luteoviridae* to the family *Solemoviridae* (order *Sobelivirales*) [15]. A characteristic *Polerovirus* genome features a covalently attached virus protein genome-linked (VPg) at the 5′-end and lacks a poly(A) tail at the 3′-end. The genome typically encodes five to seven overlapping open reading frames (ORFs), some of which are translated from subgenomic RNAs. ORF0, ORF1, and ORF2 are translated from genomic RNA, while ORF3a to ORF7 are translated from subgenomic RNA [16]. These viral proteins are primarily involved in RNA interference suppression, genome replication, virion assembly, in-plant movement, and vector transmission [17,18,19].

Poleroviruses are primarily transmitted by aphids in a circulative, non-propagative manner, causing significant economic losses in agricultural crops worldwide. The phloem-limited nature of poleroviruses restricts their movement within plants, resulting in systemic symptoms such as leaf vein yellowing, stunting, and reduced vigor, which profoundly impact crop productivity and quality [20,21]. While extensive research was conducted on poleroviruses affecting major crops, such as cereals, vegetables, and fruits, their presence and impact in traditional Chinese medicinal plants, such as *V. philippica* remain unexplored [22,23]. In this study, we utilized RNA-seq to identify viral pathogens in *V. philippica* plants exhibiting virus-like symptoms and discovered a novel virus belonging to the genus *Polerovirus,* named Viola philippica polerovirus (VPPV). Furthermore, an infectious cDNA clone of VPPV was constructed, and the virulence determinants of VPPV were investigated.

## 2. Materials and Methods

### 2.1. Plant Materials and Plant Growth

In April 2021, a *V. philippica* plant showing symptoms of yellowing, mottling, and vein chlorosis was sampled in the Summer Palace in Beijing, China. *Nicotiana benthamiana* plants were cultivated in a growth chamber maintained at 25 °C (day) and 22 °C (night), with a relative humidity of 60% and a photoperiod of 16 h (light) and 8 h (dark).

### 2.2. RNA-Seq and Data Analyses

RNA-seq and data analyses were performed as previously described [10]. Briefly, the total RNA was extracted from the leaf tissue of the collected sample using the MiniBEST Plant RNA Extraction Kit (TaKaRa, Tokyo, Japan) according to the manufacturer’s instructions. The extracted total RNA was treated with the TruSeq RNA Sample Prep Kit (Illumina, San Diego, CA, USA) to remove ribosomal RNAs. Subsequently, libraries were prepared and sequenced on an Illumina HiSeq X-ten platform (Biomarker Technologies, Beijing, China). Raw sequencing data underwent quality filtering and adapter trimming using CLC Genomics Workbench 9.5 software (QIAGEN, Germantown, MD, USA), resulting in high-quality clean reads that were de novo assembled into contigs using Trinity v2.3.2 program (Broad Institute and the Hebrew University of Jerusalem, Cambridge, MA, USA and Jerusalem, Israel). These contigs were then subjected to BLASTx and BLASTp searches in NCBI databases as described by Wang et al. [10].

### 2.3. Reverse Transcription-Polymerase Chain Reaction (RT-PCR) and 5′/3′ Rapid Amplification of cDNA Ends (5′/3′ RACE)

To obtain the full-length genome sequences of VPPV, total RNA was extracted from the collected plant samples. Reverse transcription (RT) was conducted using the ReverTra Ace qPCR RT Master Mix with gDNA Remover (Toyobo, Osaka, Japan) to generate cDNAs, followed by PCR to detect the virus in the collected plant samples using the Green Taq Mix (Vazyme, Nanjing, China) and specific primer pairs (Table S1) according to the assembled contig sequences. For the accurate amplification of viral genomic fragments, RT was performed using M-MLV reverse transcriptase (TaKaRa, Kusatsu, Japan) and fragment-specific primers, followed by super-fidelity PCR using KOD One PCR Master Mix (TOYOBO, Osaka, Japan) and specific primers (Table S2). To amplify the 3′ and 5′ ends of viral genomic RNA, RACE was performed using the HiScript-TS 5′/3′ RACE Kit (Vazyme, Nanjing, China) according to the manufacturer’s instructions. Notably, Poly(U) polymerase (NEB) was used to add a poly(A) tail to 3′ end of viral RNA genome, followed by RT using M-MLV and an oligo-dT-adapter primer to generate cDNAs. Subsequently, nested-PCR amplification was performed using gene-specific primers (GSPs) and adapter primers (Table S3). The amplified products of the expected size were gel-purified and cloned into the pCE2 TA/Blunt-Zero vector (Vazyme) for Sanger sequencing. The resulting sequences were checked and used to assemble the complete viral RNAs.

### 2.4. Organization Analysis of Viral Genome and Prediction of Viral Proteins

For open reading frame (ORF) prediction, the viral genomic sequences were analyzed using the ORFfinder service (http://www.ncbi.nlm.nih.gov/orffinder, accessed on 25 January 2021). Amino acid sequences of the predicted viral proteins were then searched against the GenBank database using BLASTp. Conserved protein domains within the deduced viral proteins were identified utilizing the Conserved Domain Search Service (CD-Search) software available through NCBI (https://www.ncbi.nlm.nih.gov/Structure/cdd/wrpsb.cgi, accessed on 9 December 2024). Genomic and protein sequences of closely related viruses were obtained from the GenBank database at NCBI (Table S4) and aligned with VPPV genomic and protein sequences using DNASTAR7.1 software (https://www.dnastar.com/, accessed on 9 December 2024).

### 2.5. Recombination and Phylogenetic Analysis

To assess viral recombination, the complete genomic sequences of VPPV and closely related viruses, including chickpea chlorotic stunt virus (CpCSV, AY956384.1), pepper vein yellows virus (PeVYV, KU999109.1), melon aphid-borne yellows virus (MABYV, EU000534.1), potato leafroll virus (PLRV, OQ446811.1), cotton leafroll dwarf virus (CLDV, GU167940.1), and cereal yellow dwarf virus (CYDV, AF235168.2), were aligned using clustalW, followed by recombination analysis conducted with the RDP5 program (http://web.cbio.uct.ac.za/~darren/rdp.html, accessed on 9 December 2024), as described by Martin et al. [24]. To elucidate, the evolutionary relationship of the virus, the amino acid sequences of RdRps or CP from VPPV, and closely related poleroviruses obtained from the GenBank database were aligned using clustalW. Subsequently, phylogenetic trees were subsequently constructed using MEGA X software (https://www.megasoftware.net/, accessed on 9 December 2024). Detailed sequence information for this study is provided in Table S4.

### 2.6. Plasmid Construction

The complete genomic sequence of VPPV was amplified by RT-PCR and cloned into the linear expression vector pCB301-RZ using MonCloneTM Hi-Fusion Cloning Mix V2 (Monad, Suzhou, China) following the manufacturer’s instructions. The viral protein genes were individually amplified from infected *V. philippica* plants via RT-PCR using specific primers listed in Table S5. The amplified products were subcloned into the linear expression vectors pGD or pGR106 utilizing MonCloneTM Single Assembly Cloning Mix (Monad, Suzhou, China) or MonCloneTM Hi-Fusion Cloning Mix V2 (Monad, Suzhou, China), according to the manufacturer’s instructions.

### 2.7. Agroinfiltration Assays

The recombinant vectors pGD or pCB301-RZ were introduced into *Agrobacterium tumefaciens* strain EHA105, while the pGR106-based vectors were individually introduced into *A. tumefaciens* strain GV3101 using the freeze-thawing procedure. The transformed *A. tumefaciens* cultures were diluted to OD600 = 0.6 with infiltration buffer (10 mM MgCl_2_, 10 mM MES, pH 5.6, and 100 μM acetosyringone) and then infiltrated into the *N. benthamiana* leaves.

### 2.8. Protein Extraction and Western Blot Assays

Plant leaves were ground to a fine powder in liquid nitrogen and homogenized in extraction buffer (50 mM Tris-HCl, pH 6.8, 9 M carbamide, 4.5% SDS, and 7.5% 2-mercaptoethanol). The resulting protein samples were subjected to electrophoresis on a 12% sodium dodecyl sulfate-polyacrylamide gel and subsequently transferred onto nitrocellulose membranes. The membranes were blocked with 5% skimmed milk in a 0.01 M phosphate-buffered saline (PBS) for 30 min at 37 °C, followed by incubation in anti-PVX-CP mouse monoclonal antibody for 60 min at 37 °C. After three rinses in PBS containing 0.05% Tween-20, the membranes were incubated in a goat anti-mouse secondary antibody conjugated horseradish peroxidase for another 60 min at 37 °C. The resulting nitrocellulose membranes were analyzed using ECL substrate and photographed by ImageQuant LAS 4000mini (GE HealthCare, New York, NY, USA).

## 3. Results

### 3.1. Identification of a Novel Polerovirus in Viola philippica

**Figure 1:**
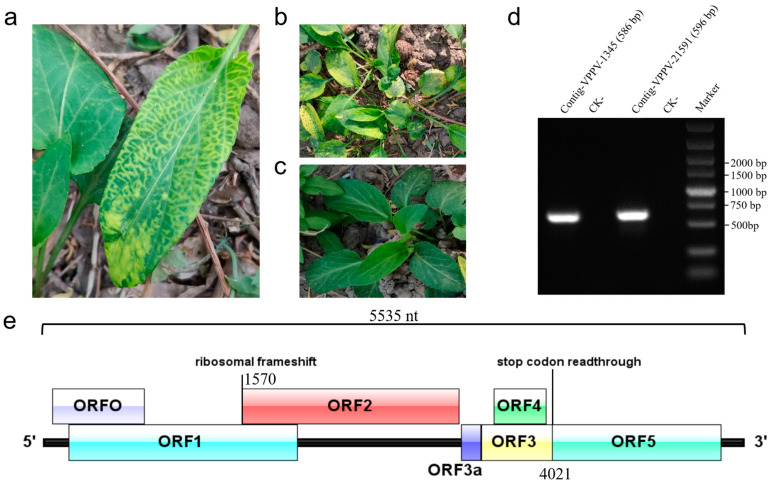
Symptoms of the diseased *Viola philippica* plants, RT-PCR detection of VPPV contigs and characteristics of the VPPV genome. (**a**,**b**) The symptomatic *V. philippica* plants show symptoms of leaf yellowing, mottling, and vein chlorosis. (**c**) Healthy *V. philippica* plants. (**d**) RT-PCR detection of contig 1345 and contig 21,591 in the symptomatic *V. philippica* plants and healthy plants (CK-). (**e**) Schematic diagram of the genome organization of VPPV. The positions of −1 ribosomal frameshift site in ORF1 and stop codon readthrough are marked in the schematic diagram.

A *V. philippica* plant exhibiting symptoms of leaf vein yellowing, mottling, and vein chlorosis was sampled (Figure 1a,b). Total RNA was extracted and subjected to RNA-seq analysis to identify the viral pathogen(s) responsible for the observed symptoms. The de novo assembly of high-quality clean reads was performed, followed by the screening of contigs longer than 1000 bp by using BLASTx algorithms. The results identified two contigs with high sequence similarity to polerovirus: contig 1345 (4044 nt) and contig 21,591 (1465 nt). To validate the RNA-seq findings, RT-PCR assays using contig-specific primers (Table S1) successfully amplified segments from both contigs (586 bp and 596 bp) in the symptomatic sample (Figure 1a) but not from asymptomatic leaf (Figure 1c). Sanger sequencing confirmed the presence of contigs 1345 and 21,591 in the symptomatic *V. philippica* sample.

Based on the genome organization of known poleroviruses (Figure 1e), we speculated that contig 1345 represents the upstream portion of the novel viral genome, while contig 21,591 corresponds to the downstream portion, with an unidentified sequence gap between these two contigs. To obtain the complete viral genome, 5′ and 3′ RACE and super-fidelity RT-PCR amplification were separately conducted to determine the terminal sequences and fill the sequence gap between these two contigs. The results revealed that the novel virus has a positive-sense, single-stranded RNA genome of 5535 nt in length, including a 69-nt 5′ UTR that begins with the conserved sequence ACAAAAGA [25], and a 179-nt 3′ UTR, excluding the poly(A) tail (Figure 1e). Genome sequence alignment analysis demonstrated that this novel virus shares 36.7% to 56.1% genome identity with twelve known viruses in the genus *Polerovirus*, with the highest identity observed with chickpea chlorotic stunt virus (CpCSV) (Table 1). The complete genome sequence of the novel virus was deposited in Genbank under accession number PP770488 and was designated as viola philippica polerovirus (VPPV).

### 3.2. Genome Organization of VPPV

The VPPV genome contains seven putative ORFs, labeled ORF0 to ORF5 and ORF3a, which are predicted to encode proteins P0 to P5 and P3a. ORF0 (nt positions 70–795) is predicted to encode a 26.9 kDa RNA silencing suppressor protein (P0), which shares the highest amino acid sequence identity (24.2%) with P0 of pepper vein yellows virus 4 (PeVYV-4) (Table 1). The ORF1 (nt positions 197–2002) encodes a 66.1 kDa polyprotein (P1), exhibiting amino acid sequence identity ranging from 26.3% to 39.1% with P1 proteins from other known poleroviruses (Table 1). P1 is predicted to contain a conserved Peptidase_S39 superfamily domain (cl09540, 215–414 aa), which is involved in protein cleavage activity.

Given that ORF1 contains a conserved slippery heptanucleotide sequence ^1564^GGGAAAC^1570^, it is anticipated that a fusion protein, i.e., RdRp weighing 115.5 kDa, is produced via a −1 ribosomal frameshift between ORF1 and ORF2 (nt positions 197–1570 and 1570–3282). The RdRp features a conserved ps-ssRNAv_RdRp-like super family domain (cl40470, 552–1007 aa), which is essential for viral replication. VPPV RdRp shares amino acid sequence identity ranging from 44.2% to 56.2% with RdRps from twelve representative poleroviruses, with the highest identity observed with the RdRp of melon aphid-borne yellows virus (MABYV) (Table 1).

ORF3 (nt positions 3461–4021) encodes a putative 21.2 kDa coat protein (CP; P3), which shares amino acid sequence identity ranging from 30.9% to 37.2% with CPs from known poleroviruses (Table 1). P3 is predicted to contain a Luteo_coat superfamily domain (cl03007, 53–180 aa). Between the translation initiation sites of RdRp and CP, there is a 178-nt intergenic region. It is hypothesized that the non-AUG-initiated protein P3a begins at nt position 3305 and terminates at a UAA stop codon at nt position 3457.

ORF4 (nt positions 3561–3971), which overlaps with ORF3 but is in a different reading frame, is predicted to encode a movement protein (MP; P4) of 15.1 kDa. VPPV P4 shares the highest amino acid identity (26.6%) with P4 of potato leafroll virus (PLRV) (Table 1). ORF5 (nt positions 3461–5356) is translated via an in-frame readthrough of the ORF3 stop codon, encoding a minor capsid component of 71.0 kDa, also referred to as the read through protein (RTP). VPPV RTP shares amino acid identity ranging from 21.5% to 30.0% with P5 of other known poleroviruses (Table 1).

### 3.3. Recombination and Phylogenetic Analysis

**Figure 2:**
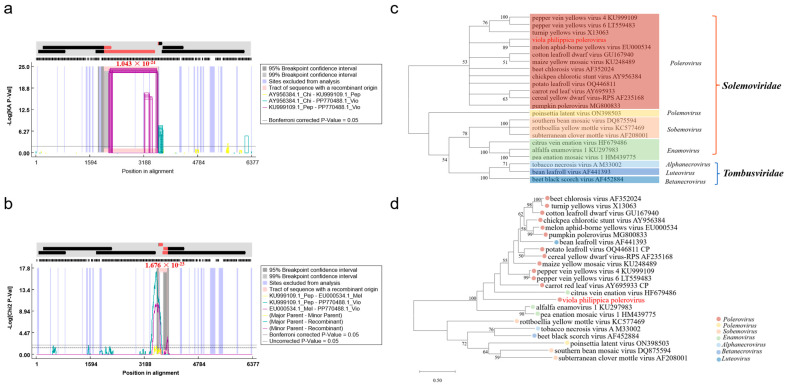
Recombination and phylogenetic analysis of VPPV with other related poleroviruses in the family *Solemoviridae* and *Tombusviridae*. Results of the recombination analyzed by GENECONV method (**a**) and MaxChi method (**b**). Lines indicate the percentage of similarity per alignment, and the pink area indicates the recombinant part of the sequence. The phylogenetic trees were constructed based on RdRp (**c**) or CP protein (**d**) using the maximum likelihood method with 1000 bootstraps. VPPV is shown in red. The bootstrap values are indicated adjacent to the nodes. Detailed sequence information of these viruses is shown in Table S4.

Poleroviruses exhibit a propensity for recombination both within their own genus and with viruses from other genera. To assess the frequency and distribution of potential recombination events in VPPV, we aligned genome sequences of VPPV (PP770488.1), CpCSV (AY956384.1), PeVYV (KU999109.1), MABYV (EU000534.1), PLRV (OQ446811.1), CLDV (GU167940.1), and CYDV (AF235168.2) using the recombination program RDP5 [24]. A putative recombination event in VPPV, with CpCSV as the major parent and PeVYV as the minor parent, was strongly supported by the GENECONV method, with *p*-values of 1.043 × 10^−24^ (Figure 2a). The predicted beginning and ending breakpoints are located at nt positions 2020 and 3622 in the VPPV genome. This region is a known “hot spot” for recombination events in poleroviruses [26]. The recombinant fragment extends from the end of ORF1 to the non-coding intergenic region (IR) of the VPPV sub-genome and encodes the C-terminal part of RdRp (P1-P2) (Figure 2a). Another putative recombination event, spanning nt positions 3620–3874 within the VPPV genome, involves PeVYV as the major parent and CpCSV or MABYV as the minor parents. This event was strongly supported by the MaxChi (*p*-values: 1.676 × 10^−23^), Chimaera (*p*-values: 6.575 × 10^−5^), and 3Seq methods (*p*-values: 3.819 × 10^−14^) (Figure 2b). This recombinant fragment spans from ORF3a to ORF3 and encodes the near-complete P3a protein and the N-terminal part of the CP (P3a-P3) (Figure 2b).

To investigate the evolutionary relationship between VPPV and other members of the family *Solemoviridae*, we aligned the amino acid sequences of RdRp or CP proteins from 19 representative viruses across different genera within the family *Solemoviridae*, alongside with 3 viruses from the family *Tombusviridae*, to construct a phylogenetic tree (Table S4). The phylogenetic tree based on RdRp clearly indicated that VPPV clustered with twelve representative poleroviruses and exhibited the closest genetic relationship with MABYV (Figure 2c), suggesting its classification as a member of the *Polerovirus* genus. Additionally, the phylogenetic tree based on viral CP further supported this assignment of VPPV to the *Polerovirus* genus (Figure 2d).

### 3.4. Construction of the Infectious cDNA Clone of VPPV

**Figure 3:**
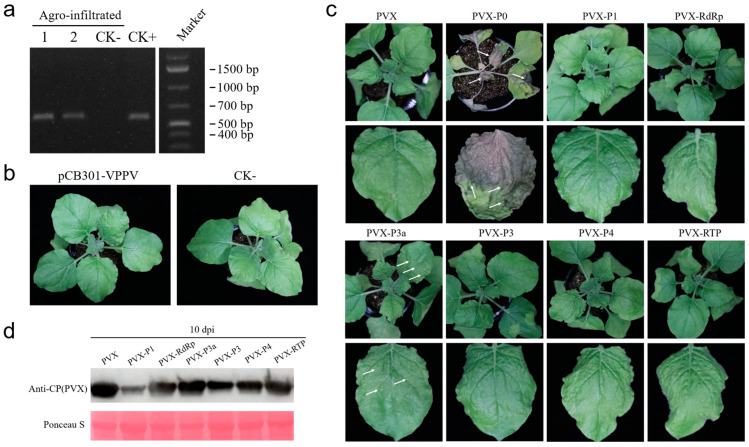
Agroinfiltration inoculation of the established VPPV infectious cDNA clone and identification of pathogenicity determinants of VPPV. (**a**) RT-PCR detection of VPPV in the systemic leaves of *N. benthamiana* plants infiltrated with *A. tumefaciens* culture carrying the pCB301-VPPV (line 1 and 2) or carrying the empty pCB301 vector (CK-). pCB301-VPPV plasmid was used as PCR positive control (CK+). (**b**) Symptoms of *N. benthamiana* plants infiltrated with *A. tumefaciens* culture carrying the pCB301-VPPV vector (**left**) or carrying the empty pCB301 vector (the CK- control, **right**). (**c**) Symptoms of *N. benthamiana* plants expressing VPPV P0, P1, RdRp, P3a, P3, P4, or RTP using PVX heterologous expression system. White arrows indicate necrotic patches. (**d**) Western blot analysis of PVX CP accumulation in systemic leaves of PVX inoculated *N. benthamiana* at 10 days post inoculation (dpi). The Rubisco stained by Ponceau S was used as the loading control.

To gain insights into virus biology, an infectious clone derived from a complete viral genome is an important tool. The full-length cDNA of VPPV was cloned into the binary vector pCB301 (pCB301-VPPV), which features the cauliflower mosaic virus 35S promoter to generate capped VPPV viral transcripts, and the autolytic hepatitis delta virus ribozyme (HDV Rz) to release the authentic VPPV viral 3′ end. *N. benthamiana* plants were infiltrated with *A. tumefaciens* harboring pCB301-VPPV or the empty pCB301 vector. At 12 days post infiltration (dpi), newly emerging leaves from the infiltrated *N. benthamiana* plants were collected and subjected to RT-PCR using VPPV-specific primer pairs (Table S1). RT-PCR products of the expected size (~586 bp) were detected in the systemic leaves of pCB301-VPPV infiltrated plants, while no products were detected in the negative control pCB301-infiltrated (CK-) plants (Figure 3a). This confirmed successful viral RNA amplification and VPPV infection in pCB301-VPPV-infiltrated *N. benthamiana* plants. However, despite the establishment of a stable viral infection, no discernible virus-like symptoms were observed in the pCB301-VPPV-infiltrated *N. benthamiana* plants (Figure 3b), suggesting that VPPV cause asymptomatic infection in its non-natural host, *N. benthamiana*.

### 3.5. Virulence Determinants of VPPV

Since the infectious cDNA clone of VPPV causes asymptomatic infection in *N. benthamiana*, a potato X virus (PVX)-based heterologous system was employed to better understand the pathology of this novel polerovirus and identify its viral virulence determinants. Seven PVX-based recombinant vectors were constructed: PVX-P0, PVX-P1, PVX-RdRp, PVX-P3a, PVX-P3, PVX-P4, and PVX-RTP. Notably, an AUG start codon was added before the putative start codon (AUA) of P3a to initiate non-canonical P3a expression. Additionally, the ORF3 stop codon (UGA) was mutated to GCG (Ala) to enable full-length ORF5 expression of the fusion protein RTP. *A. tumefaciens* carrying these expression vectors was individually infiltrated into *N. benthamiana* leaves, with the empty PVX vector serving as a negative control for comparison.

At 3 days post-infiltration (dpi), plants inoculated with PVX-P0 developed severe necrotic lesions on the infiltrated leaves. By 5 dpi, necrotic symptoms extended to the upper systemic leaves, ultimately leading to severe wilting and death of the plant (Figure 3c). These results indicate that VPPV P0 can elicit a hypersensitive response (HR) in *N. benthamiana*. At 10 dpi, PVX-P3a-infected plants developed punctate necrosis on the leaves, while plants infiltrated with PVX-RdRp, PVX-P3, PVX-P4, or PVX-RTP displayed only curling and mild mosaic symptoms, similar to those caused by negative control PVX infection (Figure 3c).

To confirm PVX infection and measure PVX accumulation in the infiltrated plants, we performed a Western blot assay using an anti-PVX coat protein (CP) antibody at 10 dpi (Figure 3d). The results confirmed successful infection by all PVX recombinant vectors in infiltrated *N. benthamiana*. However, due to the severe necrosis induced by PVX-P0, protein extraction from the PVX-P0 sample was unsuccessful (Figure 3d). Interestingly, PVX-P1 failed to induce any typical PVX-associated symptoms, such as leaf curling and mosaic patterns, throughout the entire observation period (Figure 3c). This lack of symptoms was consistent with our PVX accumulation assay, which showed significantly reduced PVX CP accumulation in PVX-P1 samples compared to the negative control (Figure 3d). Based on these results, we concluded that P0 and P3a are likely the pathogenicity determinants of VPPV.

## 4. Discussion

*V. philippica* has attracted considerable attention in the traditional Chinese medicine market due to its medicinal properties [1,2]. However, in natural ecosystems, *V. philippica* is not only susceptible to microbial pathogens but also exposed to a variety of plant-specific viruses, which pose a detrimental impact on the Chinese medicine planting industry. To date, no reports have documented viral infections in *V. philippica* plants. In this study, we employed RNA-seq analysis to investigate the presence of emerging viruses in herbaceous *V. philippica* exhibiting typical viral symptoms, such as leaf yellowing, mottling, and vein chlorosis. Our analysis led to the identification of a novel polerovirus, VPPV, and the determination of its complete genome sequence. To our knowledge, this is the first report of a novel polerovirus infecting *V. philippica*. According to the latest species demarcation criteria for the *Polerovirus* genus outlined by International Committee on Taxonomy of Viruses (ICTV), a difference in amino acid sequence identity greater than 10% in any gene product is required for species classification [14]. Our finding indicated that VPPV qualifies as a distinct species within the *Polerovirus* genus based on its viral genomic structure (Figure 1e), amino acid homology (Table 1), and phylogenetic tree analysis (Figure 2c,d).

Viruses in the economically important *Polerovirus* genus are known for their complex interactions with both their plant hosts and insect vectors [27,28]. The VPPV genomic RNA encodes three proteins, P0, P1, and RdRp, while its subgenomic RNA encodes four proteins: CP, MP, RTP, and a short non-AUG-initiated P3a. The polerovirus P0 protein is a viral suppressor of RNA silencing, targeting the ARGONAUTE1(AGO1) PAZ motif and its adjacent upstream sequence for degradation [29]. Furthermore, it was demonstrated that RNA silencing suppressors heterologous expressed by PVX can induce hypersensitive response [30,31]. Consistent with previous findings, the heterologous expression of the VPPV P0 protein by PVX resulted in plant necrosis (Figure 3c), suggesting that P0 is a key pathogenicity determinant, acting as a viral suppressor of RNA silencing and eliciting a systemic HR-like response in PVX-associated synergism.

The polyprotein (P1) in poleroviruses is translated into at least two forms (P1 and P1/P2 transframe protein)and contains a serine proteinase domain, serving as a precursor for VPg generation [32]. Further investigation is needed to clarify the self-cleavage activity and processed products of VPPV P1. The expression of VPPV P1 in heterologous PVX system mitigated typical PVX-associated symptoms (Figure 3c) and reduced PVX CP accumulation (Figure 3d), possibly due to its nucleic acid-binding properties, which negatively impact PVX replication. This is consistent with previous findings that PLRV P1/2 and P1 possess sequence-unrelated binding domains involved in PLRV RNA replication [33]. The RdRp (P1/2) of poleroviruses is a translational fusion protein generated via a -1 ribosomal frameshift mechanism [34,35]. Based on these findings, VPPV RdRp is speculated to be produced by ORF1 and ORF2 undergoing a −1 ribosomal frameshift at the slippery sequence ^1564^GGGAAAC^1570^.

The non-AUG-initiated P3a of polerovirus, encoded by ORF3a, is essential for viral long-distance movement and phloemrestriction [19]. We speculate that VPPV P3a may play a crucial role in intercellular viral movement and systemic viral infection in plants. The expression of VPPV P3a by PVX did not significantly alter the mild mosaic symptoms typically observed in PVX-infected *N. benthamiana* plants. However, occasional small scattered bleached spots were observed (Figure 3c), suggesting that this non-canonical-initiated viral protein may have subtle effects on the symptom phenotype.

In accordance with other poleroviruses, the coding region of the VPPV genomic ORF3 contains a P4, which is encoded by an alternative reading frame (Figure 1e) and is potentially associated with viral cell-to-cell movement [36]. ORF5 is expressed exclusively through an in-frame readthrough of the stop codon in ORF3 (Figure 1e), resulting in the production of RTP, a fusion protein with a C-terminus extension of CP [37,38]. VPPV RTP may facilitate viral transport through aphid salivary glands membranes, as poleroviruses and luteoviruses carrying mutations within RTP are not aphid transmissible [39,40,41].

Poleroviruses exhibit frequent intra- and interspecific recombination, particularly in regions associated with ORF2 and ORF3a [42,43]. Our findings reveal two potential recombination events concentrated at the boundaries of RdRp, P3a, and CP genes in VPPV (Figure 2a,b). One recombination event was observed in RdRp, with CpCSV identified as the major parent (Figure 2a). Another recombination event occurred in P3a and the N-terminal of CP, with PeVYV as the major parent (Figure 2b). These findings strongly suggest distinct evolutionary histories for these three genes in VPPV. This is further supported by significant incongruence between the phylogenetic trees of the RdRp and CP gene (Figure 2c,d). The clustering of recombination breakpoints in these three genes may be due to certain RdRp-P3a-CP combinations and confer a selective advantage in specific host/vector combinations [44]. These results provide valuable insights into the genetic diversity and evolution of poleroviruses.

Understanding the biology, epidemiology, and pathogenic mechanisms of poleroviruses is critical for developing effective management strategies. We successfully constructed a full-length infectious cDNA clone of VPPV and found that VPPV can asymptomatically infect *N. benthamiana* (Figure 3a,b). However, this infectivity assay should be further confirmed in its native host plant, *V. philippica*. The VPPV infectious cDNA clone will serve as a tool to investigate important viral biological traits, such as aphid vector interactions, susceptible hosts, the epidemiology, and impacts of this virus on the productivity of *V. philippica*. Furthermore, the role of *V. philippica* as a potential virus reservoir raises critical questions about the transmission pathways of poleroviruses and their impact on both wild and cultivated plants in shared environments. Understanding the interaction between *V. philippica* and poleroviruses will provide broader insights into viral epidemiology, evolution, and the development of plant virus resistance strategies.

## 5. Conclusions

This study identified and characterized a novel polerovirus, VPPV, which infects the medicinal herb *V. philippica*. A full-length infectious cDNA clone of VPPV was developed, and the P0 and P3a proteins were determined as two key pathogenic determinants. The discovery of VPPV infection in *V. philippica* advances our understanding of polerovirus diversity and evolutionary dynamics, laying the groundwork for future studies on its impact and management strategies in traditional Chinese medicinal plants.

## Figures and Tables

**Table 1:** The nucleotide and amino acid identities between VPPV and the 12 representative viruses in the genus *Polerovirus*.

Virus Name	Nucleotide Identities (%)	Amino Acid Identities (%)					
		P0	P1	RdRp	P3 (CP)	P4 (MP)	RTP
potato leafroll virus	46.5	15.8	29.2	47.6	34.2	26.6	23.8
pepper vein yellows virus 4	45.3	24.2	35.1	53.5	32.3	15.0	21.5
pepper vein yellows virus 6	36.7	23.2	35.7	54.1	33.9	15.0	26.8
beet chlorosis virus	47.9	19.8	29.6	49.5	35.9	24.6	27.2
carrot red leaf virus	47.7	16.5	30.4	50.1	34.2	20.0	23.6
cereal yellow dwarf virus-RPS	46.5	16.7	29.3	44.5	37.2	22.0	28.7
chickpea chlorotic stunt virus	56.1	21.7	37.7	49.3	35.0	23.9	29.1
cotton leafroll dwarf virus	50.5	19.2	36.8	52.3	35.5	23.1	30.0
maize yellow mosaic virus	49.1	18.0	32.2	49.4	30.9	18.0	28.3
melon aphid-borne yellows virus	51.8	23.3	39.1	56.2	35.7	22.6	29.6
pumpkin polerovirus	48.9	23.2	26.3	44.2	34.1	21.6	29.1
turnip yellows virus	50.8	22.1	37.5	54.2	36.4	25.4	27.8

Red numbers indicate the highest identity.

## Data Availability

The data generated and analyzed in this study are available upon request.

## Supplementary Materials

The following supporting information can be downloaded at: www.mdpi.com/article/10.3390/v17010114/s1, Table S1. Specific primers used to detect the VPPV contigs; Table S2. Specific primers used for RT-RCR amplification of VPPV genome sequence; Table S3. Specific primers used to perform RACE; Table S4. Virus information used to analyze the nucleotide and amino acid identities, recombination, and construct phylogenetic trees; Table S5. Specific primers used to construct vectors.
