# Supplementary material for: Genomic, Evolutionary, and Pathogenic Characterization of a New Polerovirus in Traditional Chinese Medicine Viola philippica
Source: Viruses. 2025 Jan 15;17(1):114. doi: 10.3390/v17010114 (PMC11768663; doi:10.3390/v17010114)
Supplement: Supplementary file 1 [file viruses-17-00114-s001.zip › viruses-3389030-supplementary.pdf]

Supplementary materials:

Supplementary Tables:

Table S1. Specific primers used to detect the VPPV contigs

Contig name	Primer name	Gene sequence (5′-3′)	Amplified gene length (bp)
1345	Pol-DF1	TTGGGTGTCCACATTGGGAG	586
	Pol-DR1	TGTTTTTGGGTGGGGCGATA	
21591	Pol-DF2	TGTGTACGTTGAGGCTTGCT	596
	Pol-DR2	GAACCCAAGGGTCAGGATCG	

Table S2. Specific primers used for RT-RCR amplification of VPPV genome sequence

Primer name	Gene sequence (5′-3′)	Amplified gene length(bp)
Pol-F1	ATGCAATTTTACATTGTAAAC-GAAAGG	3902
Pol-R1	CTACATCGTTCTGCGATCCGC	
Pol-F2	CCTCTGCAGGCGTCATCAAG	1260
Pol-R2	GAACCCAAGGGTCAGGATCG	
Pol-F3	CTCCTCCCCCACCTCCTCC	1311
Pol-R3	CCGGAGGGGTTTATTTTCCTATGG	

Table S3. Specific primers used to perform RACE

Primer name	Gene sequence (5′-3′)
Pol-5′GSP-1	TCAAGAAGTCCTGAAAACCACGCACGGC
Pol-5′GSP-2	CTTCCGTGGAAGTCTTCGCGGAGGAG
Pol-3′GSP-1	ATCTTTCCTATCGACAGCCGAGCGCG
Pol-3′GSP-2	AATAAACCCCTCCGGGCATAGTGTCCCC

Table S4. Viruses information used to analyze the nucleotide and amino acid identities, recombination, and construct phylogenetic trees

Virus name	Genus	Accession No.
potato leafroll virus	*Polerovirus*	OQ446811
pepper vein yellows virus 4	*Polerovirus*	KU999109
pepper vein yellows virus 6	*Polerovirus*	LT559483
beet chlorosis virus	*Polerovirus*	AF352024

carrot red leaf virus	*Polerovirus*	AY695933
cereal yellow dwarf virus-RPS	*Polerovirus*	AF235168
chickpea chlorotic stunt virus	*Polerovirus*	AY956384
cotton leafroll dwarf virus	*Polerovirus*	GU167940
maize yellow mosaic virus	*Polerovirus*	KU248489
melon aphid-borne yellows virus	*Polerovirus*	EU000534
pumpkin polerovirus	*Polerovirus*	MG800833
turnip yellows virus	*Polerovirus*	X13063
southern bean mosaic virus	*Sobemovirus*	DQ875594
rottboellia yellow mottle virus	*Sobemovirus*	KC577469
subterranean clover mottle virus	*Sobemovirus*	AF208001
alfalfa enamovirus 1	*Enamovirus*	KU297983
citrus vein enation virus	*Enamovirus*	HF679486
pea enation mosaic virus 1	*Enamovirus*	HM439775
poinsettia latent virus	*Polemovirus*	ON398503
tobacco necrosis virus A	*Alphanecrovirus*	M33002
beet black scorch virus	*Betanecrovirus*	AF452884
bean leafroll virus	*Luteovirus*	AF441393

Table S5. Specific primers used to construct vectors

Vectors	Primer name	Gene sequence (5′-3′)
PVX-P0	P0-F	tcagcaccagctagcatcgatATGCAATTTTACATTGTAAACGAAAGG
	P0-R	aaccgttcatcggcggtcgacTTAAGGCTCCTCCATAAAACCG
PVX-P1	P1-F	tcagcaccagctagcatcgatATGACGAGAATGCTGTTTCTCG
	P1-R	aaccgttcatcggcggtcgacTCAGTTCGGCTTGGGGGC
PVX-RdRp	RdRp-F1	tcagcaccagctagcatcgatATGACGAGAATGCTGTTTCTCG
	RdRp-R1	CTTCCCCCGGTTTCCCGACTGGGACGG
	RdRp-F2	AGTCGGGAAACCGGGGGAAGCGGCAGCGA
	RdRp-R2	aaccgttcatcggcggtcgacCTATGCGAGATTGTTTTGTGGC
PVX-P3a	P3a-F2	tcagcaccagctagcatcgatATGTCCAAAATTGCTCCAAATTATATC
	P3a-R2	aaccgttcatcggcggtcgacTTAACCCCTGCCGTATTCGTTG
PVX-P3	P3-F	tcagcaccagctagcatcgatATGGTCGCCCGAAGGAGG
	P3-R	aaccgttcatcggcggtcgacTCAGATTTTGTCAAAGTAGTCGACG
PVX-P4	P4-F	tcagcaccagctagcatcgatATGCAAGAAGAATACCAGCTAACAGG
	P4-R	aaccgttcatcggcggtcgacCTACATCGTTCTGCGATCCG
PVX-RTP	P5-F1	tcagcaccagctagcatcgatATGGTCGCCCGAAGGAGG
	P5-R1	ATCGCGCGCGATTTTGTCAAAGTAGTCG
	P5-F2	TTGACAAAATCGCGCGCGATGGGGGATCCCGA
	P5-R2	aaccgttcatcggcggtcgacTTATTTTCCTATGGAATCTAGAAACCAGC
pCB301-VPPV	VPPV-F1	tttcatttggagaggACAAAAGAAATCCGAGAGTGAGAG
	VPPV-R1	GGTCAATGTATGCTCCGCCGG
	VPPV-F2	GAGCATACATTGACCTTGATATCTC
	VPPV-R2	CCGGAGGGGTTTATTTTCCTATGG
	VPPV-F3	AATAAACCCCTCCGGGCATAGTGTCCCC
	VPPV-R3	atgccatgccgacccAACAGAGAAACATAGGGGTCGC
